# Supplementary material for: Analysis of a 180-degree U-turn maneuver executed by a hipposiderid bat
Source: PLoS One. 2020 Nov 3;15(11):e0241489. doi: 10.1371/journal.pone.0241489 (PMC7608926; doi:10.1371/journal.pone.0241489)
Supplement: Attachment — Submitted filename: Author_response.pdf [file pone.0241489.s004.pdf]

Author reply:

1. The authors pointed that "certain unsteady mechanisms such as enhanced lift by the leading edge vortex (LEV) have been shown to be nearly ubiquitous in flapping flight, while other mechanisms such as clap and fling are only observed in certain insects operating at smaller length scales" on page 9. I think it is better to also point that bats can dynamically changing wingspan to enhance lift (Wang et al. J R Soc Interface, 12(113), 20150821,2015; Physics of Fluids, 26(6), 061903, 2014; Physics of Fluids, 27(6), 061901, 2015)

Author Reply: We have added the reference per the suggestion.

2. The authors use 44 cells per wing chord length in the simulations. The required number of cells in numerical simulations depends strongly on the Reynolds number of the flows. What is the typical Reynolds number in the simulations?

Author Reply: The Reynolds number varies with flight velocity (as the bat is slowing down during the u-turn), but ranges from 4,000 to 10,000. We have mentioned this in the text.

3. What is the initial flow condition for the simulations?

Author Reply: The initial flow condition is zero absolute velocity (since the bat is flying in stagnant air in the flight tunnel), and we allow several time steps for the flow to develop. Since the reference frame is moving, the velocity field was initialized at the reference frame velocity. We have mentioned this in the text.

4. I suggest the authors to provide the details of the definition of force coefficients in Figure 7.

Author Reply: We have added this definition.
