# Supplementary material for: Analysis of a 180-degree U-turn maneuver executed by a hipposiderid bat
Source: PLoS One. 2020 Nov 3;15(11):e0241489. doi: 10.1371/journal.pone.0241489 (PMC7608926; doi:10.1371/journal.pone.0241489)
Supplement: Attachment — Submitted filename: author_response2.docx [file pone.0241489.s005.docx]

Thank you for revising your work. I have checked it, and would like to request a minor revision regarding the definitions of Reynolds number and lift coefficient. In 3.3, the lift coefficient is defined by using "the mean flight velocity". I think this reference velocity can be used in the definition of Reynolds number. By the way, it is better to use "lift coefficient" instead of "force coefficient" in 3.3.

1. We have been more precise in defining the Reynolds number in the text. We are now using the same $U_{\infty}$ which is used for calculating the force coefficients (initial approach velocity).

“The Reynolds number of the flight defined with respect to the initial approach velocity and the mean chord is 10,000.”

2. We have changed the nomenclature from C_L to C to define a general force coefficient because the coefficient is not only used to non-dimensionlize the lift but other directional forces as well.

“The force coefficients, $C_{x}, C_{y},$ and $C_{z}$ are defined per standard convention as,

$$C=\frac{F}{\frac{1}{2}\rho{U_{\infty}}^{2}A}$$

where $F$is the directional force in the body-fixed system $(F_{x},F_{y},{\mathrm{or} F}_{z})$, $\rho$ is the air density, $U_{\infty}$ is the initial approach flight velocity, and $A$ is the maximum planform area of the wing during the downstroke.”
